# Predicting the Development of Type 2 Diabetes in a Large Australian Cohort Using Machine-Learning Techniques: Longitudinal Survey Study

**DOI:** 10.2196/16850

**Published:** 2020-07-28

**Authors:** Lei Zhang, Xianwen Shang, Subhashaan Sreedharan, Xixi Yan, Jianbin Liu, Stuart Keel, Jinrong Wu, Wei Peng, Mingguang He

**Affiliations:** 1 China-Australia Joint Research Center for Infectious Diseases School of Public Health Xi’an Jiaotong University Health Science Center Xi'an, Shaanxi China; 2 Centre for Eye Research Australia; Ophthalmology Department of Surgery The University of Melbourne Melbourne Australia; 3 Research Centre for Data Analytics and Cognition La Trobe University Melbourne Australia

**Keywords:** diabetes, machine learning, risk prediction, cohort study

## Abstract

**Background:**

Previous conventional models for the prediction of diabetes could be updated by incorporating the increasing amount of health data available and new risk prediction methodology.

**Objective:**

We aimed to develop a substantially improved diabetes risk prediction model using sophisticated machine-learning algorithms based on a large retrospective population cohort of over 230,000 people who were enrolled in the study during 2006-2017.

**Methods:**

We collected demographic, medical, behavioral, and incidence data for type 2 diabetes mellitus (T2DM) in over 236,684 diabetes-free participants recruited from the 45 and Up Study. We predicted and compared the risk of diabetes onset in these participants at 3, 5, 7, and 10 years based on three machine-learning approaches and the conventional regression model.

**Results:**

Overall, 6.05% (14,313/236,684) of the participants developed T2DM during an average 8.8-year follow-up period. The 10-year diabetes incidence in men was 8.30% (8.08%-8.49%), which was significantly higher (odds ratio 1.37, 95% CI 1.32-1.41) than that in women at 6.20% (6.00%-6.40%). The incidence of T2DM was doubled in individuals with obesity (men: 17.78% [17.05%-18.43%]; women: 14.59% [13.99%-15.17%]) compared with that of nonobese individuals. The gradient boosting machine model showed the best performance among the four models (area under the curve of 79% in 3-year prediction and 75% in 10-year prediction). All machine-learning models predicted BMI as the most significant factor contributing to diabetes onset, which explained 12%-50% of the variance in the prediction of diabetes. The model predicted that if BMI in obese and overweight participants could be hypothetically reduced to a healthy range, the 10-year probability of diabetes onset would be significantly reduced from 8.3% to 2.8% (*P*<.001).

**Conclusions:**

A one-time self-reported survey can accurately predict the risk of diabetes using a machine-learning approach. Achieving a healthy BMI can significantly reduce the risk of developing T2DM.

## Introduction

Diabetes and its complications are major causes of premature mortality globally. It is estimated that 451 million people worldwide had diabetes in 2017, and this figure is projected to rise by 35% to 693 million by 2045 [1]. In addition to the disease burden of diabetes, the annual global economic cost associated with diabetes is currently estimated to be US $1.3 trillion [2].

Predicting the risk of diabetes in adults has been a primary focus in many health care systems internationally. In the last 20 years, numerous diabetes risk prediction tools have been developed with variable success [3-12]. Among these, four were published by national government agencies (United States [10], Australia [11], United Kingdom [9], and Canada [8]) and are freely accessible online. The vast majority of these tools collect information on individual demographical characteristics, medical history, family history, anthropometric measurements, and biomarkers, and produce a “risk score” based on regression models. However, these conventional models share some major shortcomings. First, all of these tools include blood glucose level as a predictor, which leads to spurious inflated prediction accuracy because the glucose level per se defines diabetes. Prediction based on a predicting factor that defines outcomes will inevitably achieve high accuracy. Second, these tools have been developed based on relatively small sample sizes (typically 5200-6400 individuals) and include participants recruited from only select communities. Third, the datasets utilized are outdated and therefore represent a potential source of bias. For example, the American Diabetes Association Questionnaire is based on the National Health and Nutrition Examination conducted during 1999-2004 [10] and the Australian Type 2 Diabetes Risk Assessment Tool is based on the 1999-2000 AusDiab-Australian Diabetes, Obesity and Lifestyle study [11]. Fourth, all of these tools employed a conventional regression model for risk prediction.

Therefore, these models could be updated by incorporating the increasing amount of health data available and new risk prediction methodology available to date. Interestingly, the 2014 EPIC-InterACT study reviewed and validated 12 conventional prediction models based on a case-cohort sample of 27,779 European individuals [12]. The results suggested that these models can identify individuals at high risk of developing type 2 diabetes mellitus (T2DM), but the performance of the models varied substantially with country, age, sex, and body weight. More recently, the QDiabetes study led by Hippisley-Cox et al [13] overcame many of these shortcomings. Based on a large population dataset of 11.5 million individuals, this model provides a 10-year risk prediction for diabetes with the option to include or exclude fasting blood glucose and glycated hemoglobin as predictors. Despite this progress, the study employed a conventional Cox proportional hazards model, which suffers from some major limitations associated with its assumptions in which the predictors are assumed to have time-independent and linear impacts on the hazard.

Machine learning is an emerging and widely accepted approach for risk prediction [14]. Various machine-learning algorithms have been proposed, ranging from conventional to more advanced ensemble machine-learning approaches [15]. However, a shared common trait in most models is reliance on the presence of biomarkers. For instance, the blood glucose level is a biomarker that is commonly adopted in several machine-learning models with an estimated area under the receiver operating characteristic curve (AUC) value in the 70%-80% range [16-18]. Combining the information of both blood glucose levels and other biological parameters has been shown to improve the machine-learning accuracy [19], but the collection of biomarkers requires invasive blood sampling and is limited to clinical settings. Therefore, development of an accurate prediction tool that solely depends on self-reported information offers great potential for wider application in resource-limited settings to combat the growing global diabetes epidemic.

We argue that a new risk prediction tool is needed to address the shortcomings of current tools. Toward this end, in this study, we present a machine learning–based diabetes risk prediction tool using only self-reported information. This model was based on data from a large cohort of more than 230,000 residents in New South Wales (NSW), Australia collected during the period of 2006-2017. More specifically, the tool aims to address two questions. First, can the risk of diabetes be predicted in both the short and long term (3-10 years) based on a one-time self-reported survey without any biomarkers? Second, can the effects of modifiable risk factors for diabetes onset be assessed with such a tool?

## Methods

### The 45 and Up Study

The Sax Institute’s 45 and Up Study is the largest prospective cohort study conducted in Australia [20]. This study enrolled 266,896 residents aged 45 years and older from NSW, Australia between 2006 and 2009, representing around 11% of the NSW population in this age group [20]. The study methodology has been described in detail elsewhere [20]. Eligible participants aged 45 and over and residents of NSW were randomly sampled from the Medicare Australia enrolment database, and received an invitation by mail including a study questionnaire and a written informed consent form. All participants provided consent for linkage of their information to routine health databases. The baseline questionnaire captured information on a broad range of socioeconomic, health, and lifestyle factors. To track medical procedures and medications received by the participants, the 45 and Up Study data was linked to the Medicare Benefits Schedule and Pharmaceutical Benefits Scheme claims from 2004 to 2016 using a unique identifier provided by the Department of Human Services. The Medicare Benefits Schedule code is a unique identifying code for medical procedures, whereas the Pharmaceutical Benefits Scheme is the identifying code for medications prescribed by clinicians.

### Ethical Considerations

Ethics approval of the 45 and Up Study was obtained from the University of New South Wales Human Research Ethics Committee. Approval to use data from the 45 and Up Study for the current study was received from the Royal Victorian Eye and Ear Hospital Human Research Ethics Committee.

### Inclusion and Exclusion Criteria

We excluded participants with established diabetes at baseline, defined as those who: (1) provided a positive response to question no. 24 “Has a doctor EVER told you that you have diabetes?”; (2) used diabetes medications based on the Pharmaceutical Benefits Scheme database before the baseline survey [21]; or (3) had gestational diabetes, defined as a diagnosis of diabetes earlier than the last childbirth, but without diabetes medication use subsequently. We also excluded participants who had incomplete physical activity data, and those who reported an age of diabetes diagnosis older than the age at the baseline survey. Among the 266,896 participants from the 45 and Up Study, we included a total of 236,584 residents in this study (Multimedia Appendix 1).

### Key Outcome and Predicting Variables

The primary outcome of the study was the first occurrence of prescription for any kind of medication for T2DM (including oral hypoglycemic agents and insulin). Prescription of a diabetes medication was defined as the corresponding Pharmaceutical Benefits Scheme codes detailed in Multimedia Appendix 2. As all participants were aged >45 years, we assumed that all cases of new diabetes medication use were for T2DM rather than type 1 diabetes mellitus. We intended to project the risk of diabetes with a one-time self-reported survey at baseline (Multimedia Appendix 3), which included no biomarkers such as blood glucose levels. The four categories of a total of 39 predicting variables included: demographic characteristics, medical and family history, lifestyle indicators, and dietary indicators. We acknowledge that our definition of T2DM may likely overlook cases of gestational diabetes.

### Conventional Regression Model

We employed a conventional logistic regression model to investigate the incidence of diabetes and its association with the predicting variables. We investigated the risk of diabetes and its associated factors for a duration of 3, 5, 7, and 10 years after baseline using four separate models. For each of these models, only participants who were part of the respective follow-up duration were included. We used the conventional regression model as the benchmark model as it is well established to be the standard method for investigating associations between a binary outcome and potential relevant factors.

### Machine-Learning Models

For comparison with the regression model, we applied three commonly used machine-learning models, which included a random forest, multilayer feedforward artificial neural network implementing a deep-learning approach, and a gradient boosting machine approach. These three models represent the mainstream machine-learning models for risk prediction. The random forest algorithm [22] is a supervised learning algorithm constructing an ensemble of decision trees. In this study, we used the Gini index [23] to determine the best predictive variable and location for each tree split in our algorithm. We used a cost complexity parameter to penalize more complex trees and controlled the size of the final tree. The optimal value of the complexity parameter was determined using 5-fold cross-validation. The deep-learning approach is based on the construction of an artificial neural network [24,25], and we trained this method end-to-end by stochastic gradient descent with back propagation. Gradient boosting machines employ a boosting ensemble method by minimizing an exponential loss function of the misclassification rate [26]. Gradient boosting machine performs optimization in the function space by seeking the learner (eg, decision tree) with the maximal negative gradient for the loss function [27,28].

The dataset was iterated 500 times in the model (500 epochs for deep learning, and 500 decision trees for the random forest and gradient boosting machine). A range of values for each hyperparameter was specified and all possible combinations of the hyperparameters were examined; the combination with the highest cross-validation performance metric was obtained. The random forest includes hyperparameters specifying the number of trees and the maximum depth of each tree. The parameters for deep learning included activation, hidden layer size, L1 and L2 regularization, and input dropout ratio as hyperparameters. For gradient boosting machine, a grid search for model optimization was conducted with the maximum number of models, maximum depth of each tree, learning rate, row sample rate per tree, and column sample rate as hyperparameters.

We randomly selected 70% of the total participants to form the training dataset and the remaining 30% were treated as a testing dataset. The training dataset was used for machine learning while the testing dataset was used for assessment of prediction performance of the fully trained classifiers. Five-fold cross-validation was conducted based on the training dataset.

### Model Comparisons

The AUC value was adopted to evaluate the performance of the logistic regression and machine-learning models at the predefined time points (3, 5, 7, 10 years). AUC is a robust benchmark model comparison metric for classification models, quantifying the probability of a classifier to differentiate a random positive observation over a random negative observation. The root mean square error was used to verify the result. All analyses were performed using R 3.4.1 statistical software (R Foundation for Statistical Computing, Vienna, Austria), with machine learning toolbox h2o v 3.16.0.2 (H2O.ai Inc, CA, USA). We ranked the top 10 strongest contributing factors to diabetes incidence in all four models.

The relative importance of the risk factors was ranked by their contributions to the variance in the onset of diabetes. For logistic regression, the variance was equal to the squared standardized beta coefficients. For random forest, the variance was the total decrease in node impurities from splitting on the variable, averaged over all trees. For gradient boosting machine, importance was calculated and averaged for each decision tree based on the amount that each attribute split point improves the performance measure, weighted by the number of observations the node is responsible for. For deep learning, importance was determined by identifying all weighted connections between the nodes of interest.

### Model Prediction

We used the most accurate (highest AUC value) validated model to identify the potential reduction in the probability of diabetes onset by assuming hypothetical changes in participants’ BMI categories. We investigated three scenarios: (1) all individuals in the “obese” BMI category (≥30) became “overweight” (BMI=25.0-29.9); (2) in addition to scenario 1, all individuals in the “overweight” BMI category moved to the “healthy” BMI (18.5-24.9) category; and (3) all individuals in the “obese” and “overweight” BMI categories moved to the “healthy” BMI category.

## Results

### Participant Characteristics

The baseline demographic characteristics of the study population are summarized in Multimedia Appendix 3. In brief, of the 236,684 individuals included in this retrospective cohort study, approximately 6.05% (14,313/236,684) developed T2DM during an average follow up of 8.8 years (range 7.0-11.5; 2,006,194 person years). Individuals with diabetes were significantly more likely to be older, male, overweight or obese, less educated, have a family history of diabetes, reside in a major city, and have a lower income and socioeconomic status (Chi square tests, all *P*<.0001). Further, individuals with diabetes were more likely to have self-reported hypertension, cardiovascular disease, and dyslipidemia at enrolment (all *P*<.0001). In terms of lifestyle factors, individuals with diabetes were significantly more likely to be former or current smokers, engage in less physical activity, have longer daily sitting times, consume more processed meat, and have lower milk intake (all *P*<.0001).

### Cumulative Diabetes Incidence by Gender, Age, and BMI Groups

**Figure 1:**
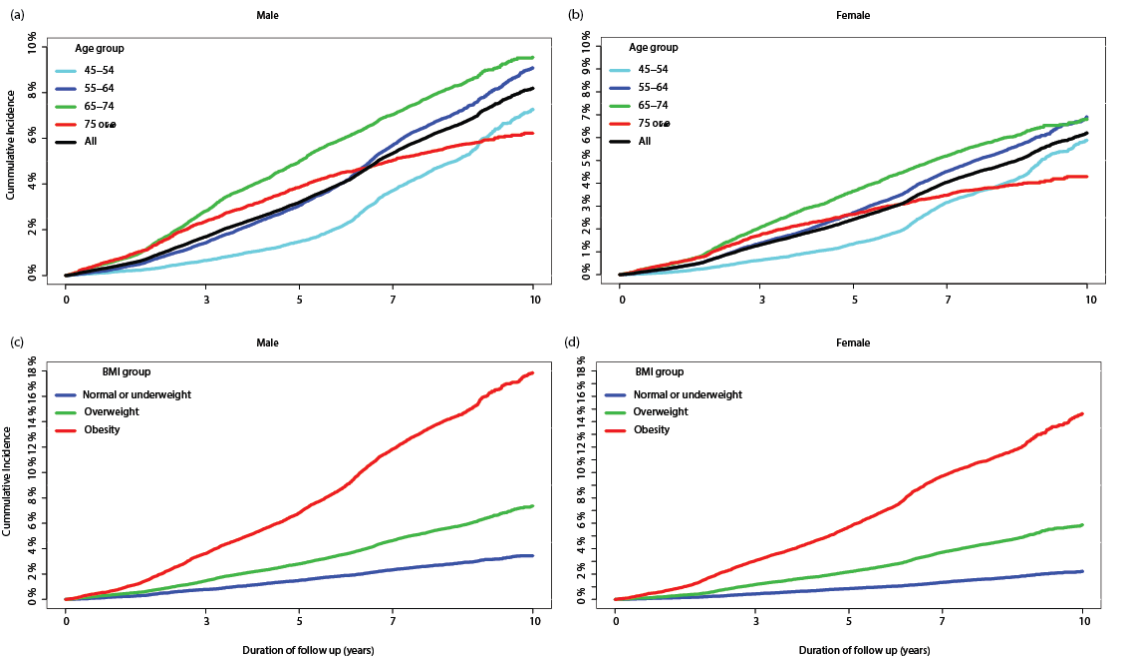
Cumulative incidence of diabetes, stratified by age groups in men and women, and stratified by BMI groups in men and women.

The cumulative incidence of diabetes was significantly higher in men than in women (Figure 1). At the end of 10 years, the cumulative diabetes incidence was 7.66% (7.23%-8.12%) in men, which was significantly higher than that of women (5.84%, range 5.49%-6.20%; odds ratio 1.37, 95% CI 1.32-1.41).

In both men and women, the age group 65-74 years had the highest cumulative incidence of diabetes (10-year incidence: 9.32%, range 8.34%-10.42%), followed by the age groups 45-54 (6.37%, range 5.67%-7.16%), 55-64 (8.68%, range 7.87%-9.57%), and ≥75 (5.84%, range 4.95%-6.88%) years. The incidence of diabetes among participants aged ≥75 years increased at a much slower rate than that of the other age groups and showed a notable reduction after 6-7 years of follow up. This occurred at a time point where the older age group approached the average life expectancy (84.6 years old) in the Australian population [29].

Men with obesity had the highest incidence of diabetes, with a 3, 5, 7, and 10 years cumulative incidence of 3.61% (3.36%-3.89%), 6.82% (6.47%-7.19%), 11.84% (11.37%-12.32%), and 17.39% (15.87%-19.05%), respectively. These were significantly higher than the cumulative incidence in men with a BMI in the overweight and healthy ranges. In particular, the 10-year diabetes incidence in men with obesity was 2.76 (2.61-2.91) and 5.83 (5.41-6.28) higher than that in overweight and healthy weight men, respectively. Diabetes incidence rates in women followed a similar pattern (Figure 1).

### Prediction of Diabetes Risk With Machine-Learning Techniques

**Figure 2:**
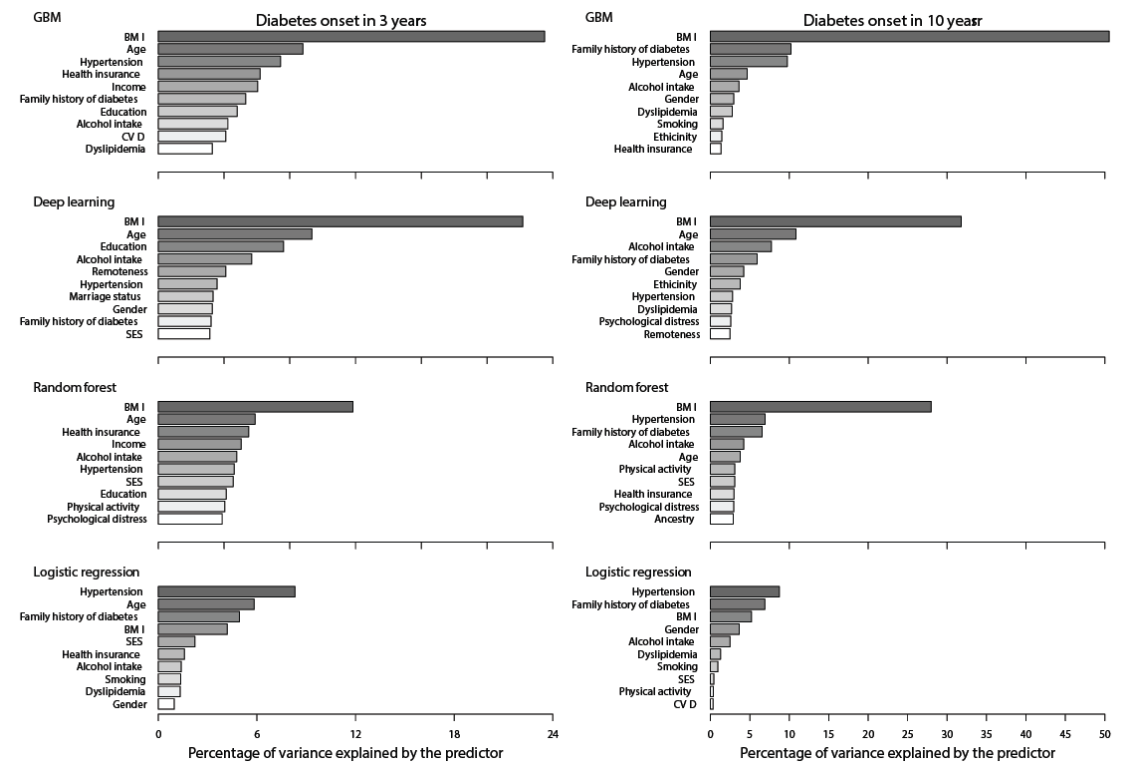
Ranked contribution to the variance of diabetes prediction by various models. (+ increasing risk; - decreasing risk; * being male increases risk compared with being female; # being born overseas increases diabetes risk compared with being born in Australia; § having private insurance decreases risk compared with having no private insurance; $ being in major cities increases risk compared with being in inner or outside regional areas; ‡ having Asian or other ancestry increases diabetes risk compared with having Australian ancestry). GBM: gradient boost machine.

Machine-learning approaches demonstrated an overall superior prediction of diabetes risk than the conventional regression analysis (Table 1). The gradient boosting machine model produced the highest accuracy of all four models for 3-year risk prediction. This was followed by the random forest and deep-learning models. Performance measured by AUC in all three machine-learning models was significantly higher than that of the regression analysis (Delong test, all *P*<.0001). A similar pattern was observed for other follow-up durations, but the power of model prediction was reduced by 5%-6% at 10-year follow up. The root mean square error was also the lowest for the gradient boosting machine model (Figure 2, Table 1).

**Table 1:** Comparison of model performance between logistic regression and machine-learning models.

Duration	Logistic regression		Gradient boosting machine		Deep learning		Random forest	
	AUC^a^ (range)	RMSE^b^	AUC (range)	RMSE	AUC (range)	RMSE	AUC (range)	RMSE
3 years	0.7401 (0.7262-0.7541)	0.1203	0.7927 (0.7803-0.8051)	0.1197	0.7769 (0.7639-0.7899)	0.1244	0.7868 (0.7742-0.7993)	0.1198
5 years	0.7192 (0.7084-0.7301)	0.1633	0.7769 (0.7673-0.7864)	0.1620	0.7610 (0.7566-0.7762)	0.1667	0.7769 (0.7612-0.7804)	0.1622
7 years	0.6990 (0.6901-0.7077)	0.2087	0.7589 (0.751-0.7668)	0.2063	0.7526 (0.7446-0.7606)	0.2099	0.7531 (0.7452-0.761)	0.2066
10 years	0.6885 (0.6801-0.6961)	0.2318	0.7491 (0.7426-0.7570 )	0.2314	0.7374 (0.7339-0.7486)	0.2435	0.7439 (0.7365-0.7510)	0.2318

^a^AUC: area under the receiver operating characteristic curve.

^b^RMSE: root mean squared error.

The machine-learning models indicated that BMI was the most important predicting factor for the occurrence of diabetes (Figure 2). In the short term (3-year follow up), all three machine-learning models consistently demonstrated that BMI alone contributed to 12%-24% of the variance in the prediction of diabetes. In contrast, BMI contributed to 20%-50% of the variance in the long term (10-year follow up).

### Prediction of Diabetes Risk Reduction

Given that BMI was the most important predictor of diabetes, we explored the potential impacts of BMI reduction on the risk of diabetes onset using the validated gradient boosting machine model. The model predicted that the probability of an obese individual developing diabetes over a 10-year period was approximately one in seven (13.4%, Table 2). In simulated scenario 1, a change of BMI level from obese to overweight significantly reduced the probability of diabetes onset to 6.2% (Table 2). Further, if both obese and overweight individuals were to improve their BMI by a single category (scenario 2), the 10-year probability of diabetes reduced from 8.3% (pooled overweight and obese subgroup) to 3.9%. A greater decline was observed when overweight and obese individuals improved their BMI to the healthy range (scenario 3), with a 10-year probability of diabetes of 2.8%.

**Table 2:** Model-predicted probability of diabetes onset in three scenarios compared with their respective status quo scenarios.

Scenario		Baseline scenario	Scenarios with hypothetical BMI change	*t* statistic (df)	*P* value
**Scenario 1^a^(N=46,645)**					
	Year 3	3.04%	1.54%	6611.97 (93,288)	<.001
	Year 5	5.81%	2.89%	7957.43 (93,288)	<.001
	Year 7	10.62%	4.68%	12,120.59 (93,288)	<.001
	Year 10	13.43%	6.22%	12,732.71 (93,288)	<.001
**Scenario 2^b^(N=133,830)**					
	Year 3	1.93%	1.02%	15,401.27 (267,658)	<.001
	Year 5	3.68%	1.94%	17,086.55 (267,658)	<.001
	Year 7	6.41%	2.98%	23,460.63 (267,658)	<.001
	Year 10	8.26%	3.93%	24,604.81 (267,658)	<.001
**Scenario 3^c^(N=133,830)**					
	Year 3	1.93%	0.77%	20,856.85 (267,658)	<.001
	Year 5	3.68%	1.50%	22,630.22 (267,658)	<.001
	Year 7	6.41%	2.14%	31,002.83 (267,658)	<.001
	Year 10	8.26%	2.79%	33,214.27 (267658)	<.001

^a^Scenario 1: “obese” individuals but become “overweight.”

^b^Scenario 2: “obese” individuals become “overweight” and “overweight” individuals reach a “healthy” BMI.

^c^Scenario 3: all “obese” and “overweight” individuals reach a “healthy” BMI.

### Model Sensitivity and Specificity

We identified the sensitivity and specificity trend versus the risk of diabetes (Multimedia Appendix 4). The trend curves were characterized by a sharp decline in sensitivity and an increase in specificity as the risk of diabetes increased. Crossing of the sensitivity and specificity represents the situation where the two indicators were equal. The model-assigned cut-off levels were consistently lower than the crossing values of the curves, indicating that the models had preferentially weighted on higher sensitivity than specificity.

## Discussion

### Principal Findings

Our study is a retrospective cohort study of more than 230,000 Australians over a follow-up period spanning a decade. Several important findings can be highlighted. First, we confirmed that machine-learning models performed significantly better than the conventional regression model in predicting the risk of diabetes onset. Notably, the models were developed based solely on self-reported information that was ascertained at a single time point but still achieved 73%-80% accuracy for diabetes prediction for up to 10 years. Second, all machine-learning models consistently demonstrated that BMI is a key risk factor contributing to the onset of T2DM.

Based on these results, we argue that a sophisticated machine-learning model is key for the risk prediction of T2DM onset. In our study, machine-learning models were demonstrated to be superior to the conventional regression model in diabetes risk prediction in a large population-based dataset. Further, the fact that our models were completely based on self-reported information in the absence of any biomarkers suggests the potential for self-assessment in individuals and primary surveillance of diabetes risk in the community. The model tracked over 230,000 Australian individuals for a duration of 10 years and is able to estimate the risk of diabetes development for each individual. Notably, the 10 strongest contributing factors explained over 74%-89% of the variation in diabetes risk. Compared with similar models that are also based on self-reported information [30,31], our model performed consistently better in predicting the risk of diabetes in both the short and long term. This provides further evidence that a simple and user-accessible self-assessment tool can be developed to project the risk of diabetes with robust accuracy, without the assistance of health care workers or need for biomarker sampling or measurement. On a population level, by using a big data platform, the collection of individual assessment surveys may inform the trends in the diabetes epidemic. This can potentially form an inexpensive user-driven online surveillance platform that surveys diabetes risk factors in a large population, which can in turn forecast the trend of the incidence of diabetes. This is potentially more advantageous than the passive hospital-based case report of diabetes diagnosis that inevitably falls behind the epidemic and population studies that are expensive and unsustainable. Our findings suggest a feasible method such as an electronic health platform for both self-assessment of diabetes risk in individuals and the monitoring of diabetes trends on a population level.

Our finding that BMI is the leading risk factor for T2DM risk was consistent across all machine-learning models. A previous study demonstrated that excessive BMI gain and earlier onset of overweight/obesity are associated with impaired glucose tolerance and diabetes onset [32]. Mokdad et al [33] further demonstrated that being overweight increases the risk of diabetes by 2 fold, while obesity increases the diabetes risk by 3-7 fold. Consistent with previous reports [34], we found that BMI alone accounted for 25%-50% of the variance in diabetes risk.

We further quantified the impact of BMI reduction on the risk of diabetes onset in several hypothetical scenarios. We predicted that reducing an individual’s BMI from “obese” to “overweight” would reduce their risk of diabetes in the short and long term by more than half. Further, if BMI could be changed from the “obese and overweight” to “healthy” range, the corresponding risk of diabetes could be reduced by almost two-thirds. This implies that interventions for diabetes prevention should prioritize weight control, especially for those in their late 60s and early 70s. According to the World Health Organization (WHO) global status report on noncommunicable diseases [35], 39% and 12.9% of adults aged 18 years or over in 2014 globally were overweight and obese, respectively, and the worldwide prevalence of obesity has doubled since 1980. Actions to address overweight and obesity are critical to preventing T2DM, as advocated in the WHO report on diabetes [2]. The WHO Global NCD Action Plan 2013–2020 listed halting the rise in diabetes and obesity as one of its voluntary global targets [36]. Our findings are in line with these WHO reports and support their key recommendations.

### Strengths and Limitations

The key strengths of the current study include the utilization of a large cohort study dataset (>230,000 participants) with a long follow-up period, and the robust performance of our algorithm for diabetes risk prediction using machine-learning models. Several study limitations should also be noted. First, the analysis was based on a large population survey with information that is subject to self-report bias. Second, the incidence of diabetes in our study was not based on the actual diagnosis of diabetes but was instead inferred by the new use of diabetes-related medications as reported in the Pharmaceutical Benefits Scheme database. This may have resulted in not identifying participants with early diabetes or prediabetes that were not on diabetic medications, and could have therefore underestimated the true diabetes incidence rate over the follow-up period. Nevertheless, one study based on 45 and Up data and linked clinical data proved that diabetes classification based on the Pharmaceutical Benefits Scheme database is more accurate than clinical data [21]. Third, questions related to eating habits in the 45 and Up Study were oversimplified and may not be comparable to standard nutritional surveys. We did not find any association between eating habits and diabetes in our study. Fourth, the absence of mortality data in our dataset implies that the T2DM risk in participants who died before its onset cannot be determined. Fifth, similar to other machine-learning algorithms, the gradient boosting machine model is likely to suffer from overfitting as it automatically removes less fit simulations during its optimization. Regularization parameters and processes such as grid search-tuned learning rate and cross-validation were utilized in this study to enhance the generality of the model. Future work will focus on further validating this model in an independent existing dataset before its official deployment.

### Conclusion

In conclusion, we have presented a sophisticated and accurate machine-learning model that allows for the prediction of T2DM incidence for up to 10 years following a single self-reported survey. The model findings highlight the significant impact of higher BMI on diabetes risk and reinforce interventions for weight control to reduce the growing prevalence of diabetes.

## Appendix

Multimedia Appendix 1 Flowchart for population selection.

Multimedia Appendix 2 List of codes for hypoglycemic medications in the Pharmaceutical Benefit Scheme.

Multimedia Appendix 3 Demographic, medical and family history, lifestyle and dietary indicators for 236,584 participants in the 45 and Up Study.

Multimedia Appendix 4 Sensitivity and specificity trend versus the risk of diabetes by the logistic regression, deep-learning, gradient boosting machine, and random forest models.

## Abbreviations

AUC: area under the curve
NSW: New South Wales
T2DM: type 2 diabetes mellitus
WHO: World Health Organization
