# Supplementary material for: Predicting the Development of Type 2 Diabetes in a Large Australian Cohort Using Machine-Learning Techniques: Longitudinal Survey Study
Source: JMIR Med Inform. 2020 Jul 28;8(7):e16850. doi: 10.2196/16850 (PMC7420582; doi:10.2196/16850)
Supplement: Multimedia Appendix 1 [file medinform_v8i7e16850_app1.docx]

Flowchart for population selection
