# Supplementary material for: Predicting the Development of Type 2 Diabetes in a Large Australian Cohort Using Machine-Learning Techniques: Longitudinal Survey Study
Source: JMIR Med Inform. 2020 Jul 28;8(7):e16850. doi: 10.2196/16850 (PMC7420582; doi:10.2196/16850)
Supplement: Multimedia Appendix 2 [file medinform_v8i7e16850_app2.docx]

**List of codes for hypoglycemic medication in Pharmaceutical Benefit Scheme.**

| **Schedule of Pharmaceutical Benefits：** | | |
| --- | --- | --- |
| **Codes for diabetes medication** | | |
| Generic name | Specifications and packaging specifications | Code |
| INSULIN ASPART | insulin aspart 100 units/mL injection, 1 x 10 mL vial | 8571D |
|  | insulin aspart 100 units/mL injection, 5 x 3 mL cartridges | 8435Y |
| INSULIN GLULISINE | insulin glulisine 100 units/mL injection, 1 x 10 mL vial | 9224L |
|  | insulin glulisine 100 units/mL injection, 5 x 3 mL cartridges | 1921D |
| INSULIN LISPRO | insulin lispro 100 units/mL injection, 5 x 3 mL cartridges | 8212F |
|  | insulin lispro 100 units/mL injection, 1 x 10 mL vial | 8084L |
| INSULIN NEUTRAL BOVINE | insulin neutral bovine 100 units/mL injection, 1 x 10 mL vial | 1713E |
| INSULIN NEUTRAL HUMAN | insulin neutral human 100 units/mL injection, 1 x 10 mL vial | 1531N |
|  | insulin neutral human 100 units/mL injection, 5 x 3 mL cartridges | 1762R |
| INSULIN ISOPHANE BOVINE | insulin isophane bovine 100 units/mL injection, 1 x 10 mL vial | 1711C |
| INSULIN ISOPHANE HUMAN | insulin isophane human 100 units/mL injection, 5 x 3 mL cartridges | 1761Q |
|  | insulin isophane human 100 units/mL injection, 1 x 10 mL vial | 1533Q |
| INSULIN ASPART + INSULIN ASPART PROTAMINE | insulin aspart 30 units/mL + insulin aspart protamine 70 units/mL injection, 5 x 3 mL syringes | 8609D |
| INSULIN ISOPHANE HUMAN + INSULIN NEUTRAL HUMAN | insulin neutral human 30 units/mL + insulin isophane human 70 units/mL injection, 1 x 10 mL vial | 1426C |
|  | insulin isophane human 70 units/mL + insulin neutral human 30 units/mL injection, 5 x 3 mL cartridges | 1763T |
|  | insulin neutral human 50 units/mL + insulin isophane human 50 units/mL injection, 5 x 3 mL cartridges | 2062M |
| INSULIN LISPRO + INSULIN LISPRO PROTAMINE | insulin lispro 50 units/mL + insulin lispro protamine 50 units/mL injection, 5 x 3 mL cartridges | 8874C |
|  | insulin lispro 25 units/mL + insulin lispro protamine 75 units/mL injection, 5 x 3 mL cartridges | 8390N |
| INSULIN DETEMIR | insulin detemir 100 units/mL injection, 5 x 3 mL cartridges | 9040T |
| INSULIN GLARGINE | insulin glargine 100 units/mL injection, 5 x 3 mL cartridges | 9039R |
| METFORMIN | metformin hydrochloride 500 mg tablet, 100 | 2430X |
|  | metformin hydrochloride 850 mg tablet, 60 | 1801T |
|  | metformin hydrochloride 1 g modified release tablet, 60 | 3439B |
|  | metformin hydrochloride 500 mg modified release tablet, 120 | 9435N |
|  | metformin hydrochloride 1 g tablet, 90 | 8607B |
| GLIBENCLAMIDE | glibenclamide 5 mg tablet, 100 | 2939Q |
| GLICLAZIDE | gliclazide 80 mg tablet, 100 | 2449X |
|  | gliclazide 60 mg modified release tablet, 60 | 9302N |
|  | gliclazide 30 mg modified release tablet, 100 | 8535F |
| GLIMEPIRIDE | glimepiride 1 mg tablet, 30 | 8450R |
|  | glimepiride 2 mg tablet, 30 | 8451T |
|  | glimepiride 4 mg tablet, 30 | 8452W |
|  | glimepiride 3 mg tablet, 30 | 8533D |
| GLIPIZIDE | glipizide 5 mg tablet, 100 | 2440K |
| ALOGLIPTIN + METFORMIN | alogliptin 12.5 mg + metformin hydrochloride 500 mg tablet, 56 | 10033C |
|  | alogliptin 12.5 mg + metformin hydrochloride 850 mg tablet, 56 | 10032B |
|  | alogliptin 12.5 mg + metformin hydrochloride 1 g tablet, 56 | 10035E |
| DAPAGLIFLOZIN + METFORMIN | dapagliflozin 10 mg + metformin hydrochloride 1 g modified release tablet, 28 | 10515K |
|  | dapagliflozin 5 mg + metformin hydrochloride 1 g modified release tablet, 56 | 10510E |
|  | dapagliflozin 10 mg + metformin hydrochloride 500 mg modified release tablet, 28 | 10516L |
| EMPAGLIFLOZIN + METFORMIN | empagliflozin 5 mg + metformin hydrochloride 1 g tablet, 60 | 10649L |
|  | empagliflozin 5 mg + metformin hydrochloride 500 mg tablet, 60 | 10650M |
|  | empagliflozin 12.5 mg + metformin hydrochloride 500 mg tablet, 60 | 10639Y |
|  | empagliflozin 12.5 mg + metformin hydrochloride 1 g tablet, 60 | 10640B |
| EMPAGLIFLOZIN + METFORMIN | empagliflozin 5 mg + metformin hydrochloride 1 g tablet, 60 | 10627H |
|  | empagliflozin 5 mg + metformin hydrochloride 500 mg tablet, 60 | 10626G |
|  | empagliflozin 12.5 mg + metformin hydrochloride 500 mg tablet, 60 | 10633P |
|  | empagliflozin 12.5 mg + metformin hydrochloride 1 g tablet, 60 | 10677Y |
| LINAGLIPTIN + METFORMIN | linagliptin 2.5 mg + metformin hydrochloride 500 mg tablet, 60 | 10038H |
|  | linagliptin 2.5 mg + metformin hydrochloride 1 g tablet, 60 | 10044P |
|  | linagliptin 2.5 mg + metformin hydrochloride 850 mg tablet, 60 | 10045Q |
| METFORMIN + GLIBENCLAMIDE | metformin hydrochloride 250 mg + glibenclamide 1.25 mg tablet, 90 | 8838E |
|  | metformin hydrochloride 500 mg + glibenclamide 2.5 mg tablet, 90 | 8810Q |
|  | metformin hydrochloride 500 mg + glibenclamide 5 mg tablet, 90 | 8811R |
| ROSIGLITAZONE + METFORMIN | rosiglitazone 4 mg + metformin hydrochloride 500 mg tablet, 56 | 9061X |
|  | rosiglitazone 4 mg + metformin hydrochloride 1 g tablet, 56 | 9062Y |
|  | rosiglitazone 2 mg + metformin hydrochloride 500 mg tablet, 56 | 9059T |
|  | rosiglitazone 2 mg + metformin hydrochloride 1 g tablet, 56 | 9060W |
| SAXAGLIPTIN + METFORMIN | saxagliptin 5 mg + metformin hydrochloride 1 g modified release tablet, 28 | 10051B |
|  | saxagliptin 5 mg + metformin hydrochloride 500 mg modified release tablet, 28 | 10055F |
|  | saxagliptin 2.5 mg + metformin hydrochloride 1 g modified release tablet, 56 | 10048W |
| SITAGLIPTIN + METFORMIN | sitagliptin 50 mg + metformin hydrochloride 850 mg tablet, 56 | 9450J |
|  | sitagliptin 50 mg + metformin hydrochloride 1 g modified release tablet, 56 | 10090C |
|  | sitagliptin 50 mg + metformin hydrochloride 1 g tablet, 56 | 9451K |
|  | sitagliptin 50 mg + metformin hydrochloride 500 mg tablet, 56 | 9449H |
|  | sitagliptin 100 mg + metformin hydrochloride 1 g tablet: modified release, 28 | 10089B |
| VILDAGLIPTIN + METFORMIN | vildagliptin 50 mg + metformin hydrochloride 850 mg tablet, 60 | 5475E |
|  | vildagliptin 50 mg + metformin hydrochloride 1 g tablet, 60 | 5476F |
|  | vildagliptin 50 mg + metformin hydrochloride 500 mg tablet, 60 | 5474D |
| ACARBOSE | acarbose 100 mg tablet, 90 | 8189B |
|  | acarbose 50 mg tablet, 90 | 8188Y |
| PIOGLITAZONE | pioglitazone 15 mg tablet, 28 | 8694N |
|  | pioglitazone 45 mg tablet, 28 | 8696Q |
|  | pioglitazone 30 mg tablet, 28 | 8695P |
| ALOGLIPTIN | alogliptin 6.25 mg tablet, 28 | 2944Y |
|  | alogliptin 12.5 mg tablet, 28 | 2933J |
|  | alogliptin 25 mg tablet, 28 | 2986E |
| LINAGLIPTIN | linagliptin 5 mg tablet, 30 | 3387G |
| SAXAGLIPTIN | saxagliptin 2.5 mg tablet, 28 | 10128C |
|  | saxagliptin 5 mg tablet, 28 | 8983T |
| SITAGLIPTIN | sitagliptin 100 mg tablet, 28 | 9182G |
|  | sitagliptin 50 mg tablet, 28 | 9181F |
|  | sitagliptin 25 mg tablet, 28 | 9180E |
| VILDAGLIPTIN | vildagliptin 50 mg tablet, 60 | 3415R |
| EXENATIDE | exenatide 2 mg/dose injection: modified release, 4 injection devices | 10888C |
| EMPAGLIFLOZIN | empagliflozin 25 mg tablet, 30 | 10202Y |
|  | empagliflozin 10 mg tablet, 30 | 10206E |
| DAPAGLIFLOZIN | dapagliflozin 10 mg tablet, 28 | 10011X |
| EXENATIDE | exenatide 5 microgram/dose injection, 60 doses | 3423E |
|  | exenatide 10 microgram/dose injection, 60 doses | 3424F |
| EXENATIDE | exenatide 2 mg/dose injection: modified release, 4 injection devices | 10888C |
| VILDAGLIPTIN | vildagliptin 50 mg tablet, 60 | 3415R |

* The version used in the present study was effective from 1 September 2017, available at the website: www.pbs.gov.au.
