# Supplementary material for: Predicting the Development of Type 2 Diabetes in a Large Australian Cohort Using Machine-Learning Techniques: Longitudinal Survey Study
Source: JMIR Med Inform. 2020 Jul 28;8(7):e16850. doi: 10.2196/16850 (PMC7420582; doi:10.2196/16850)
Supplement: Multimedia Appendix 3 [file medinform_v8i7e16850_app3.docx]

**Demographic, medical and family history, lifestyle and dietary indicators for 236,584 participants in the 45 and Up Study.**

| **Items** | **Total** | **Non-diabetes** | **Diabetes** | **P-value** |
| --- | --- | --- | --- | --- |
| **Demographic Characteristics** |  |  |  |  |
| Age group |  |  |  |  |
| *45-54* | 73838 (31.2) | 70152 (31.6) | 3686 (25.8) |  |
| *55-64* | 77682 (32.8) | 72497 (32.6) | 5185 (36.2) |  |
| *65-74* | 49575 (21.0) | 45879 (20.6) | 3696 (25.8) |  |
| ≥*75* | 35489 (15.0) | 33743 (15.2) | 1746 (12.2) | <0.0001 |
| Gender |  |  |  |  |
| *Male* | 106360 (45.0) | 98889 (44.5) | 7471 (52.2) |  |
| *Female* | 130224 (55.0) | 123382 (55.5) | 6842 (47.8) | <0.0001 |
| Annual income categories (AUD $) |  |  |  |  |
| *<20000* | 43157 (18.2) | 39839 (17.9) | 3318 (23.2) |  |
| ≥*20000-39999* | 40852 (17.3) | 38290 (17.2) | 2562 (17.9) |  |
| ≥*40000-69999* | 43132 (18.2) | 40801 (18.4) | 2331 (16.3) |  |
| ≥*70000* | 59190 (25.0) | 56406 (25.4) | 2784 (19.5) |  |
| *missing* | 50253 (21.2) | 46935 (21.1) | 3318 (23.2) | <0.0001 |
| Education level |  |  |  |  |
| *<10 years* | 25848 (10.9) | 23618 (10.6) | 2230 (15.6) |  |
| *High school/TAFE** | 150065 (63.4) | 140915 (63.4) | 9150 (63.9) |  |
| *University or higher* | 56991 (24.1) | 54324 (24.4) | 2667 (18.6) |  |
| *Missing* | 3680 (1.6) | 3414 (1.5) | 266 (1.9) | <0.0001 |
| Residential rurality |  |  |  |  |
| *Major cities* | 122870 (51.9) | 114943 (51.7) | 7927 (55.4) |  |
| *Inner regional* | 82715 (35.0) | 78113 (35.1) | 4602 (32.2) |  |
| *Outer regional and remote* | 26517 (11.2) | 24960 (11.2) | 1557 (10.9) | <0.0001 |
| *Missing* | 4482 (1.9) | 4255 (1.9) | 227 (1.6) |  |
| Insurance |  |  |  |  |
| *Private with extras* | 104985 (44.4) | 99318 (44.7) | 5667 (39.6) |  |
| *Private no extras* | 27846 (11.8) | 26544 (11.9) | 1302 (9.1) |  |
| *Health care concession* | 62059 (26.2) | 57265 (25.8) | 4794 (33.5) |  |
| *None of the above* | 37319 (15.8) | 35088 (15.8) | 2231 (15.6) | <0.0001 |
| *Missing* | 4375 (1.8) | 4056 (1.8) | 319 (2.2) |  |
| Marital status |  |  |  |  |
| *Married/Partner* | 178684 (75.5) | 167989 (75.6) | 10695 (74.7) |  |
| *Single/Widowed/Divorced* | 57900 (24.5) | 54282 (24.4) | 3618 (25.3) | 0.0196 |
| Social economic status# |  |  |  |  |
| *1^st^ quintile* | 47842 (20.2) | 44263 (19.9) | 3579 (25.0) |  |
| *2^nd^ quintile* | 48039 (20.3) | 44969 (20.2) | 3070 (21.4) |  |
| *3^rd^ quintile* | 42983 (18.2) | 40361 (18.2) | 2622 (18.3) |  |
| *4^th^ quintile* | 39251 (16.6) | 37081 (16.7) | 2170 (15.2) |  |
| *5^th^ quintile* | 52113 (22.0) | 49570 (22.3) | 2543 (17.8) | <0.0001 |
| *Missing* | 6356 (2.7) | 6027 (2.7) | 329 (2.3) |  |
| BMI |  |  |  |  |
| *Underweight and normal (≤24.9)* | 87363 (36.9) | 85363 (38.4) | 2000 (14.0) |  |
| *Overweight (25.0-29.9)* | 87185 (36.9) | 82263 (37.0) | 4922 (34.4) |  |
| *Obesity (≥30.0)* | 46645 (19.7) | 40292 (18.1) | 6353 (44.4) | <0.0001 |
| *Missing* | 15391 (6.5) | 14353 (6.5) | 1038 (7.3) |  |
|  |  |  |  |  |
| **Medical and family history** |  |  |  |  |
| Cancer at baseline |  |  |  |  |
| *No* | 200485 (84.7) | 188374 (84.7) | 12111 (84.6) |  |
| *Yes* | 36099 (15.3) | 33897 (15.3) | 2202 (15.4) | 0.6639 |
| Hypertension at baseline |  |  |  |  |
| *No* | 159038 (67.2) | 151815 (68.3) | 7223 (50.5) |  |
| *Yes* | 77546 (32.8) | 70456 (31.7) | 7090 (49.5) | <0.0001 |
| CVD at baseline |  |  |  |  |
| *No* | 207786 (87.8) | 195872 (88.1) | 11914 (83.2) |  |
| *Yes* | 28798 (12.2) | 26399 (11.9) | 2399 (16.8) | <0.0001 |
| Dyslipidemia at baseline |  |  |  |  |
| *No* | 204880 (86.6) | 193571 (87.1) | 11309 (79.0) |  |
| *Yes* | 31704 (13.4) | 28700 (12.9) | 3004 (21.0) | <0.0001 |
| Family history of diabetes |  |  |  |  |
| *No* | 187906 (79.4) | 178288 (80.2) | 9618 (67.2) |  |
| *Yes* | 48668 (20.6) | 43974 (19.8) | 4694 (32.8) | <0.0001 |
| *missing* | 10 (0.0) | 9 (0.0) | -- |  |
|  |  |  |  |  |
| **Lifestyle indicators** |  |  |  |  |
| Smoking |  |  |  |  |
| *Never smoked* | 136946 (57.9) | 129477 (58.3) | 7469 (52.2) |  |
| *Ex-smoker* | 82203 (34.7) | 76736 (34.5) | 5467 (38.2) |  |
| *Current smoker* | 17356 (7.3) | 15986 (7.2) | 1370 (9.6) | <0.0001 |
| *Missing* | 79 (0.0) | 72 (0.0) | 7 (0.0) |  |
| Number of alcohol drinks (per week) |  |  |  |  |
| 0 | 73852 (31.2) | 68210 (30.7) | 5642 (39.4) |  |
| 1-4 | 47615 (20.1) | 44816 (20.2) | 2799 (19.6) |  |
| 5-7 | 34792 (14.7) | 33123 (14.9) | 1669 (11.7) |  |
| 8-14 | 42404 (17.9) | 40397 (18.2) | 2007 (14.0) |  |
| ≥15 | 34294 (14.5) | 32365 (14.6) | 1929 (13.5) | <0.0001 |
| Missing | 3627 (1.5) | 3360 (1.5) | 267 (1.9) |  |
| Sessions of physical activities (per week) |  |  |  |  |
| <5 | 42835 (18.1) | 39516 (17.8) | 3319 (23.2) |  |
| ≥5–9 | 69529 (29.4) | 65170 (29.3) | 4359 (30.5) |  |
| ≥9–14 | 56311 (23.8) | 53284 (24.0) | 3027 (21.1) |  |
| ≥14 | 59460 (25.1) | 56440 (25.4) | 3020 (21.1) | <0.0001 |
| Missing | 8449 (3.6) | 7861 (3.5) | 588 (4.1) |  |
| Average sleeping time per day |  |  |  |  |
| <7 | 35945 (15.2) | 33318 (15.0) | 2627 (18.4) |  |
| ≥7-9 | 177508 (75.0) | 167622 (75.4) | 9886 (69.1) |  |
| ≥9 | 16738 (7.1) | 15422 (6.9) | 1316 (9.2) | 0.1685 |
| *Missing* | 6393 (2.7) | 5909 (2.7) | 484 (3.4) |  |
| Average sitting time per day |  |  |  |  |
| <8 | 164667 (69.6) | 154988 (69.7) | 9679 (67.6) |  |
| ≥8 | 71917 (30.4) | 67283 (30.3) | 4634 (32.4) | <0.0001 |
| Self-reported depression level |  |  |  |  |
| *None* | 90611 (38.3) | 85619 (38.5) | 4992 (34.9) |  |
| *Moderate* | 77224 (32.6) | 72824 (32.8) | 4400 (30.7) |  |
| *High* | 47610 (20.1) | 44117(19.8) | 3493 (24.4) | <0.0001 |
| *Missing* | 21139 (8.9) | 19711 (8.9) | 1428 (10.0) |  |
|  |  |  |  |  |
| **Dietary indicators** |  |  |  |  |
| Number of vegetable servings per day |  |  |  |  |
| <5 servings | 157088 (66.4) | 147633 (66.4) | 9455 (66.1) |  |
| ≥5 servings | 73830 (31.2) | 69458 (31.2) | 4372 (30.5) | 0.0813 |
| *Missing* | 5666 (2.4) | 5180 (2.3) | 486 (3.4) |  |
| Number of fruit servings per day |  |  |  |  |
| <2 servings | 92829 (39.2) | 86965 (39.1) | 5864 (41.0) |  |
| ≥2 servings | 131637 (55.6) | 124149 (55.9) | 7488 (52.3) | 0.0984 |
| *Missing* | 12118 (5.1) | 11157 (5.0) | 961 (6.7) |  |
| Type of breakfast |  |  |  |  |
| *High fibre* | 36397 (15.4) | 33813 (15.2) | 2584 (18.1) |  |
| *Not high fibre* | 156336 (66.1) | 147773 (66.5) | 8563 (59.8) | 0.0233 |
| *Missing* | 43851 (18.5) | 40685 (18.3) | 3166 (22.1) |  |
| Milk in breakfast |  |  |  |  |
| No milk | 12296 (5.2) | 11515 (5.2) | 781 (5.5) |  |
| Soy/skimmed/reduced fat milk | 140519 (59.4) | 132199 (59.5) | 8320 (58.1) |  |
| Full-fat /other milk | 78898 (33.3) | 74098 (33.3) | 4800 (33.5) | 0.009 |
| *Missing* | 4871 (2.1) | 4459 (2.0) | 412 (2.9) |  |
| Times of processed meat intake per week |  |  |  |  |
| 0 | 49253 (20.8) | 46835 (21.1) | 2418 (16.9) |  |
| 1-7 | 152587 (64.5) | 142944 (64.3) | 9643 (67.4) | <0.0001 |
| *Missing* | 34744 (14.7) | 32492 (14.6) | 2252 (15.7) |  |
| *TAFE stands for ‘technical and further education’, a technical institute with a similar standard a senior high school.  #Social Economic Status’s quintile cut-offs are pre-specified according to ‘Australian Bureau of Statistics. 2039.0 - Information paper: an introduction to socio-economic indexes for areas (SEIFA), 2006. Canberra 2008’. | | | | |
