# Supplementary material for: Predicting the Development of Type 2 Diabetes in a Large Australian Cohort Using Machine-Learning Techniques: Longitudinal Survey Study
Source: JMIR Med Inform. 2020 Jul 28;8(7):e16850. doi: 10.2196/16850 (PMC7420582; doi:10.2196/16850)
Supplement: Multimedia Appendix 4 [file medinform_v8i7e16850_app4.docx]

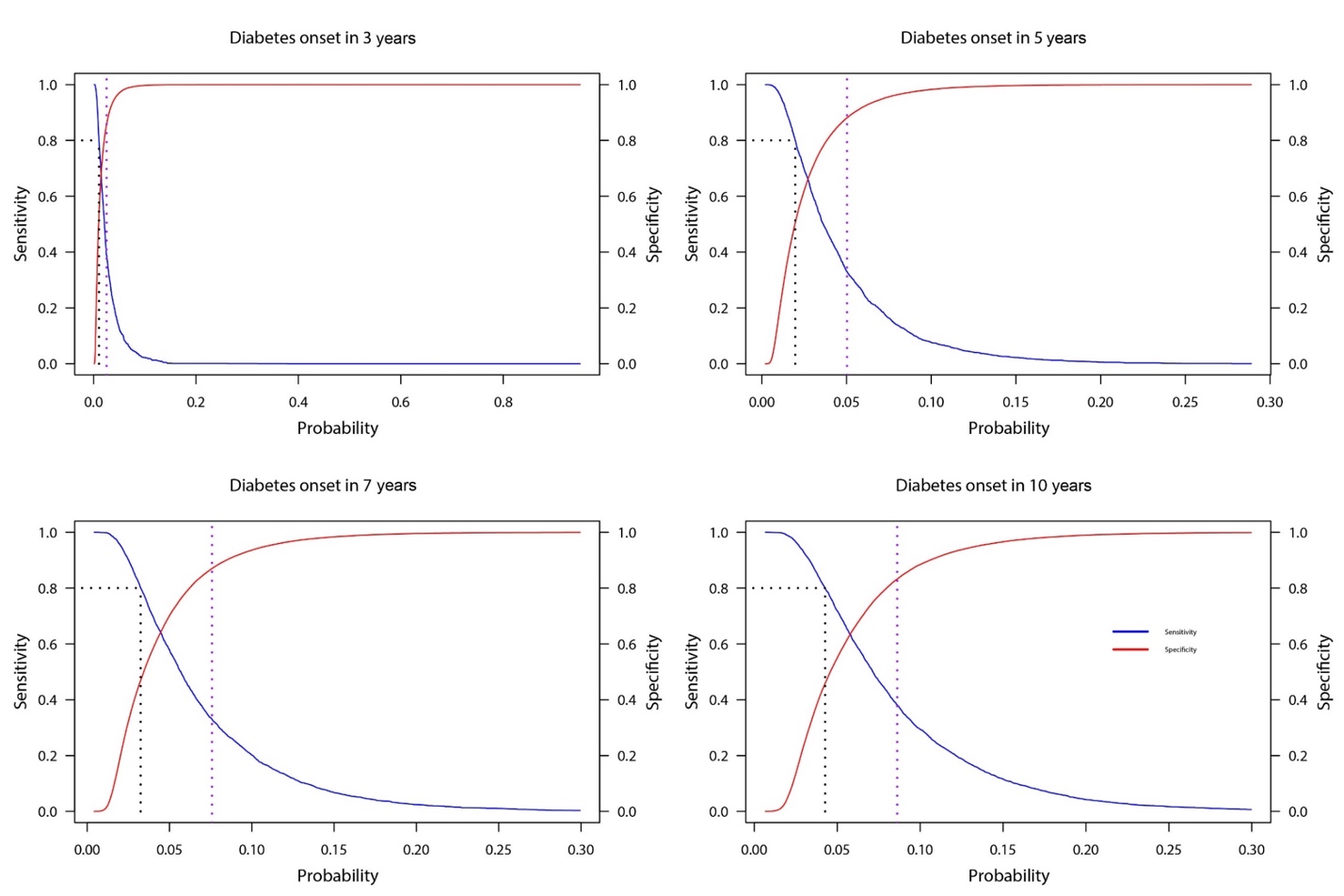


Sensitivity and specificity trend versus the risk of diabetes by the logistic regression model.


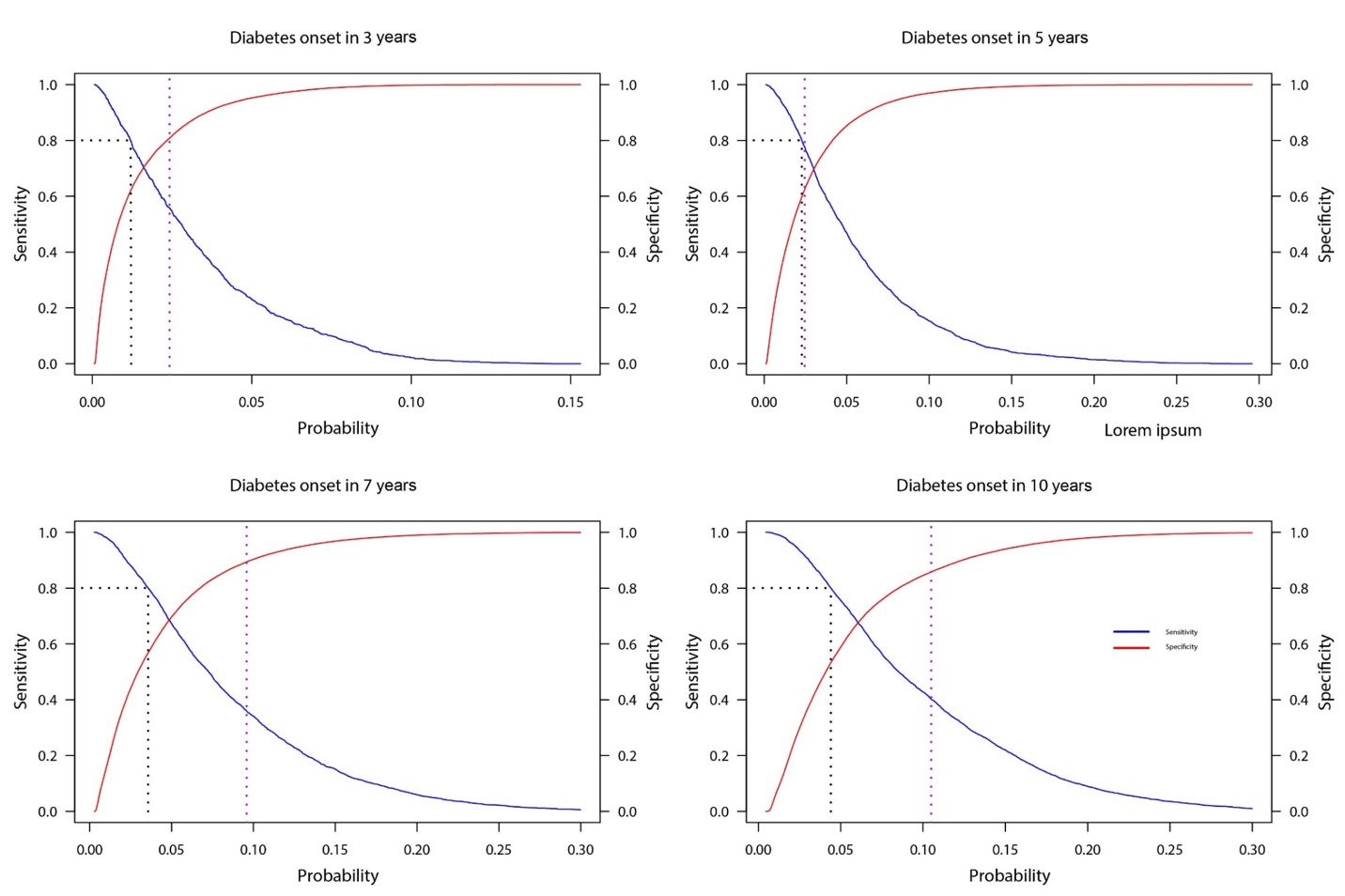


Sensitivity and specificity trend versus the risk of diabetes by the deep-learning model.


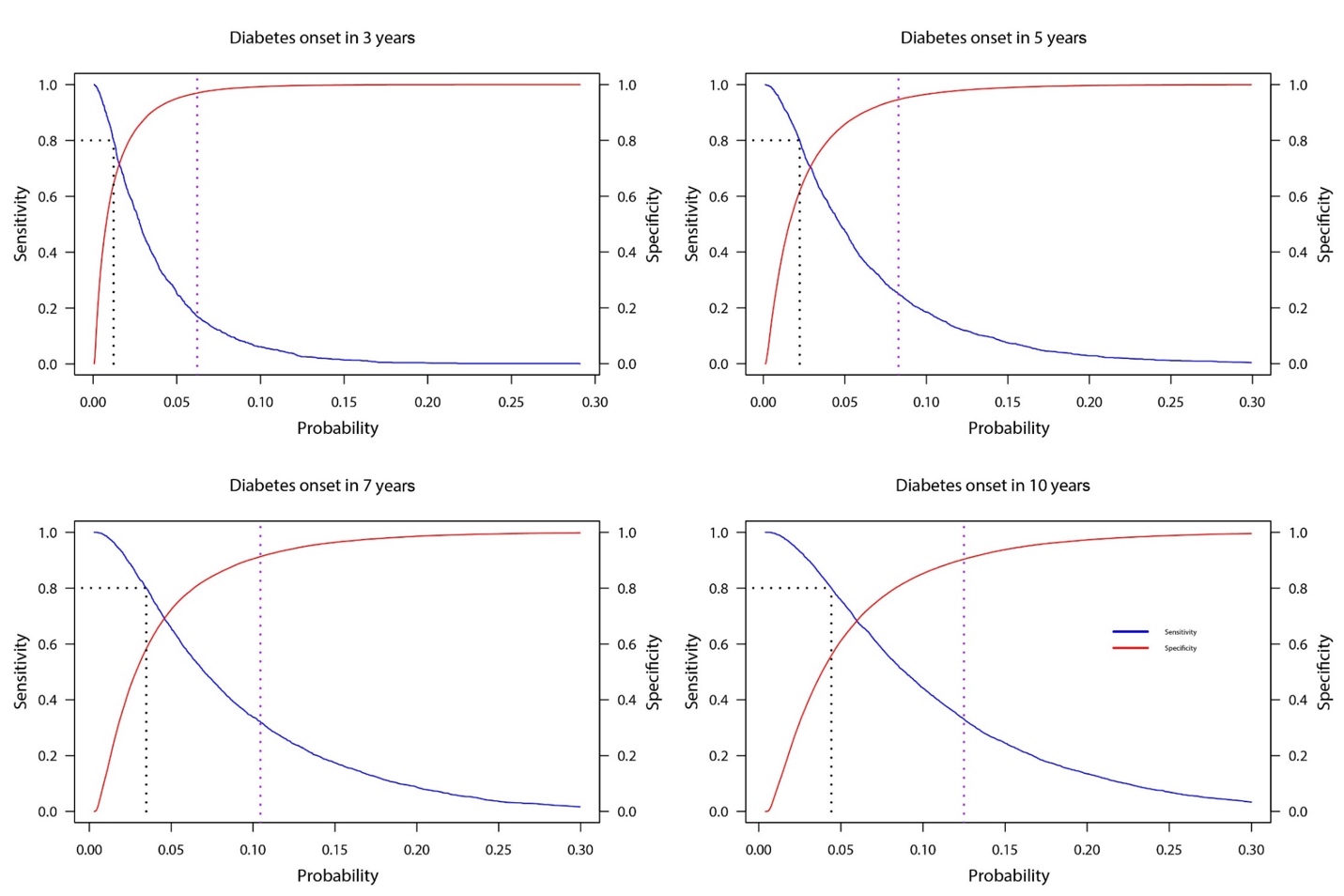


Sensitivity and specificity trend versus the risk of diabetes by the gradient boosting machines model.


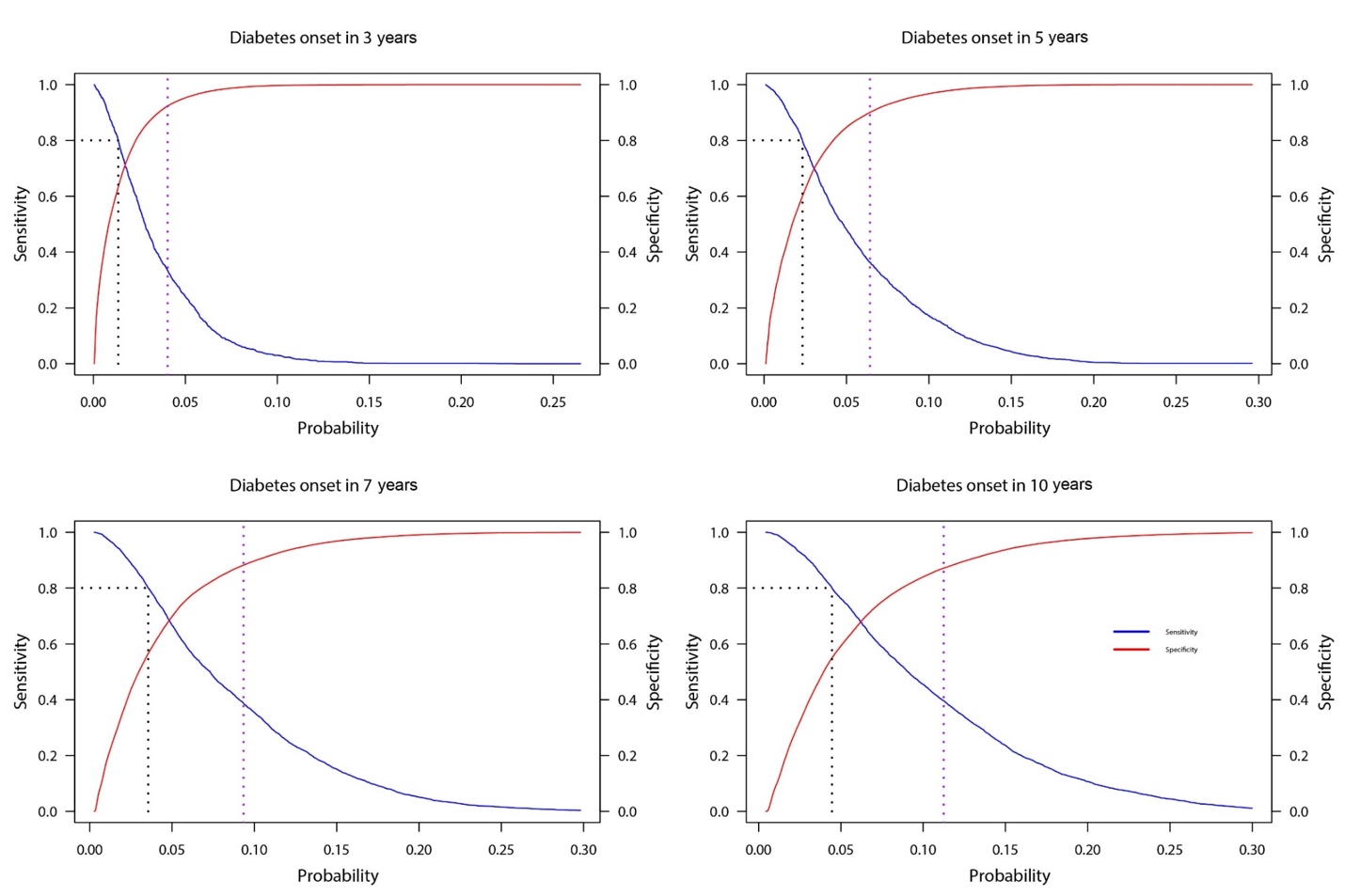


Sensitivity and specificity trend versus the risk of diabetes by the random forest model.
